# *Phytopythium vexans* Associated with Apple and Pear Decline in the Saïss Plain of Morocco

**DOI:** 10.3390/microorganisms9091916

**Published:** 2021-09-09

**Authors:** Salma Jabiri, Chaimaa Bahra, Dustin MacLean, Nabil Radouane, Essaid Ait Barka, Mohamed Bendriss Amraoui, Rachid Lahlali

**Affiliations:** 1 Phytopathology Unit, Department of Plant Protection, Ecole Nationale d’Agriculture de Meknès, B.P. S/40, Meknès 50001, Morocco; jabirisalma17@gmail.com (S.J.); cbahra@enameknes.ac.ma (C.B.); nabil.radouane@usmba.ac.ma (N.R.); 2 Faculty of Sciences Dhar El Mehraz, Sidi Mohamed Ben Abdellah University, Fès-Atlas, B.P. 1796, Fez 30050, Morocco; mohamed.bendrissamraoui@usmb.ac.ma; 3 University of British Columbia Okanagan Campus, 3333 University Way, Kelowna, BC V1V 1V7, Canada; dmacle02@uoguelph.ca; 4 Laboratory of Functional Ecology and Environmental Engineering, Sidi Mohamed Ben Abdellah University, P.O. Box 2202, Routed’Imouzzer, Fez 30050, Morocco; 5 Morocco Unité de Recherche Résistance Induite et Bio-Protection des Plantes-EA 4707, Université de Reims Champagne-Ardenne, 51100 Reims, France; ea.barka@univ-reims.fr

**Keywords:** dieback disease, *oomycetes*, sequencing, phylogeny

## Abstract

An extensive survey conducted in the Saïss plain of Morocco during the 2017–2018 growing season revealed that 35 out of 50 apple and pear orchards were infested with a pathogen that causes the decline disease. Morphological and phylogenetic tree analyses using the cox II gene allowed us to identify the pathogen as *Phytopythium vexans*. Interestingly, no *Phytophthora* and *Pythium* species were isolated. The occurrence and prevalence of the disease varied between locations; the most infested locations were Meknes (100%), Imouzzer (83%), and Sefrou (80%). To fulfill Koch’s postulate, a greenhouse pathogenicity test was performed on the stem and collar of one-year-old healthy seedlings of apple rootstock M115. Symptoms similar to those observed in the field were reproduced in less than 4 months post-inoculation with root rot disease severity ranging from 70 to 100%. The survey results evidenced that apple rootstocks, soil type, and irrigation procedure may contribute significantly to the occurrence of the disease. The disease was most prevalent in drip water irrigation and sandy-clay soil on wild apple rootstock. Accordingly, a rational drip advanced watering system and good sanitation practices could eliminate water stagnation and help prevent the onset of this disease. It was concluded that *Pp. vexans* occurrence may be strongly influenced by irrigation mode and type of soil. Therefore, the obtained findings of this study could help to better understand the recurrence of this disease and to develop a reliable integrated strategy for its management.

## 1. Introduction

Several microorganisms ubiquitous in the soil have been assumed to be causal agents of apple and pear decline diseases [1], with microorganisms belonging to the oomycetes considered to be the main causal agents of these diseases [2]. They are divided into several more or less virulent genera and species; the most devastating of which are the species belonging to *Pythium* and *Phytophthora* [3]. Root rot is the most common disease caused by these pathogens [4]. *Phytophthora* and *Pythium* species can survive for a long time in the soil and in diseased plants. They grow under persistent conditions of humidity, too frequent watering, or excessive irrigation and at temperatures around 15–16 °C. These pathogens spread through streams, watering, certain cultural practices, and by transportation of agricultural products [5,6,7]. Symptoms begin at the roots or collar; the roots are stubbed near the root collar and become rotted. In addition, the root system is reduced with the appearance of sores and rot sometimes making the roots appear brown and spongy, especially in the crown of some conifer species. These oomycetes are also involved in negative plant-soil feedback due to monoculture and replanting problems of apple orchards [8,9,10].

In Morocco, weather conditions are diversified, which allows for the production of a wide range of fruit during each growing season. The rosaceous fruit trees cover an area of more than 300,000 ha, distributed between stone fruit 85% of total production, and 15% for quince, apple pear, and almond, with the majority being almond trees [11]. The Middle Atlas, the Rif, Saïss, Haouz, and Moulouya are the main production areas, with more than 56% of the total planted area being apple trees. Apple trees occupied the largest rosaceous planted area with 32,000 ha and an estimated yield of 600,000 tons a year [12]. However, rosaceous fruit crops are largely threatened by devastating and widespread diseases in most parts of the world due to climate change, as well as changes in cultural practices [13].

A new genus of *Phytopythium*, a relative of *Phytophthora* and *Pythium* has been reported [14] as a pathogen of several fruit trees, including citrus in Tunisia [15], kiwifruit in Turkey [16], avocados in the Canary Islands [17,18], and grapevines in South Africa [19], and isolated from freshwater environments in Korea [20]. Similar symptoms to those described previously were seen in apple and pear trees in Morocco. Recently, Jabiri et al. [21] reported, for the first time, the occurrence of *Phytopythium vexans* on apple trees. Therefore, the present study will focus on determining the occurrence and distribution of these oomycete pathogens in apple and pear growing area of Saïss.

In 2018, symptoms similar to decline disease were observed on rosaceous fruit trees, including apple and pear, in growing areas of the Saïss region. The most prevalent symptoms were dry necrotic bark with brown lesions and yellowing foliage, wilting, and trunk cankers, crown and root rot causing tree decline. The role played by oomycete species in apple and pear disease in Morocco and has not yet been fully investigated. A preliminary investigation evidenced the occurrence of *Phytopythium vexans* as the major causal agent of these symptoms [21]. Therefore, an extensive survey was undertaken to better understand the etiology of the disease. The present study focuses on the distribution of the oomycete genus pathogenic to apple and pear trees in Morocco, in order to (i) identify and characterize the oomycete microorganisms associated with the disease, (ii) investigate the possible farming factors and practices allowing the occurrence of the disease, and (iii) fulfill Koch’s postulates in order to verify the role of the isolated pathogen as causative agent of the disease.

## 2. Materials and Methods

### 2.1. Sampling Sites

**Figure 1:**
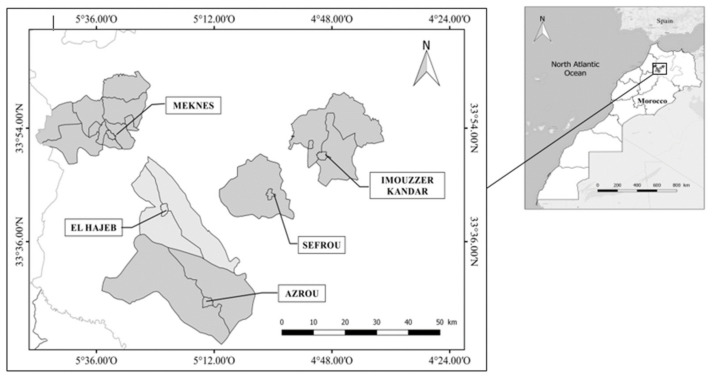
Map showing locations of the Meknes-Fes region in Morocco (Upper quadrat) and enlarged views of Meknes, El-Hajeb, Imouzzer-Kander, Sefrou, and Azrou (from the shaded square). The map was performed by ArcGIS software (v.10.9) and indicated the names of different districts where samples were collected.

Fifty orchards of apple and pear trees in five locations of the Fez-Meknes region (Meknes, El Hajeb, Sefrou, Imouzzer, and Azrou) were sampled to look for the responsible agent of tree decline (Figure 1). Most orchards were selected based on tree health information provided by producers and technical advisers of agricultural cooperatives. The criteria by which the target areas were selected were early symptoms of tree decline such as reduced growth and vigor, leaf chlorosis, or other more obvious symptoms like wilting, yellowing and defoliation, and dieback of shoots and branches.

The sampling method consisted of taking soil samples in triplicate from three different locations around each diseased tree. Each composite sample was made up of three sub-samples taken from the soil in the targeted area at a depth of 10 to 20 cm below the organic surface layer, while collecting the lateral roots. Approximately 1 kg of soil was collected by mixing sub-samples from all three sites. Each representative sample was sieved through a 5 mm sieve. Soil samples (50) were stored in a cold room until use [22].

### 2.2. The Survey

Datasets on cultural practices that may influence disease development such as soil type, cultivars, rootstocks, and irrigation systems were collected during this survey and the impact of these cultural practices was assessed. As the occurrence of oomycete pathogens is significantly influenced by watering, farmers were interviewed about drip irrigation (flow rate per tree and duration) versus submersion irrigation (delivered amount of water per tree and duration between two consecutive watering).

### 2.3. Isolation of the Pathogenic Fungi: Fruit and Soil Baiting

**Figure 2:**
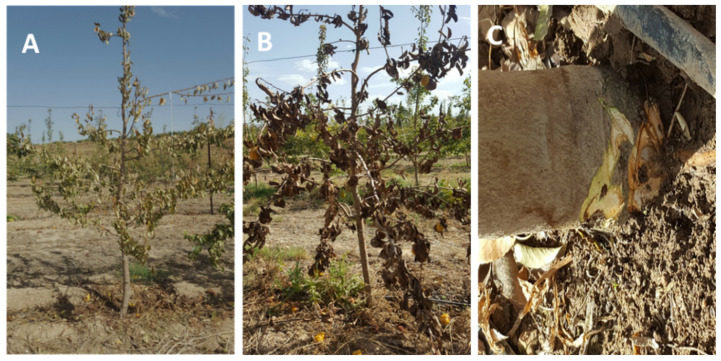
Decline disease symptoms such as yellowing (**A**), wilting (**B**), and brown lesions on the crown (**C**) on young pear tree sampled in the Sefrou location, Louata farm.

According to Jabiri et al. [21], symptoms observed on apple and pear trees in orchards (Figure 2) were caused by oomycete pathogens. To isolate these pathogens, two techniques were employed and conceived in the laboratory. The first technique consisted of using pear fruit as bait for the 50 samples. For each of the 50 samples, three plastic boxes labeled sterile were used; each (1 L capacity) was filled with soil samples on which a disinfected green pear fruit was placed, and sterile distilled water was added until the fruit was submerged. Subsequently, boxes were sealed and incubated at 4 °C in a cold chamber for 7–10 days. Pear fruit presenting brown lesions were cut into smaller pieces of 1 cm and four pieces were placed into Petri dishes containing modified CMA medium. All plates were sealed with parafilm and incubated at 25 °C in darkness. After 7 days post-incubation, fungal isolates were checked, subcultured, and purified on PDA medium.

The second technique consisted of using apple seeds as bait. Apple seeds were disinfected by soaking in a solution of 1% sodium hypochlorite (NaOCI) for 2 min, rinsed three times with sterile distilled water (SDW) and dried at ambient temperature. The seeds were shelled, cut in half, and used as live bait for oomycetes. Four cotyledons were deposited on the soil surface already prepared and placed in Petri dishes [23]. Petri dishes were filled with 60 mL of SDW. Three boxes were used for each representative soil sample. These dishes were then incubated at 4 °C and checked daily for the appearance of white mycelium on apple cotyledons. The cotyledons were examined under the microscope 4 days post-incubation, and those with coenocytic mycelium were removed and gently washed with SDW to clear cotyledons of adhering soil debris. Mycelial filaments were taken from the edge of the cotyledon using a sterile needle, then placed on Petri dishes containing modified corn meal agar (CMA) medium. To render this medium selective, a stock solution composed of the following ingredients: penicillin G (1 g), Polymyxin B (1 g), ampicillin (1 g), PCNB (pentachloronitrobenzene, 0.01 g), and hymexazol (0.5 mL) was prepared in 100 mL of SDW. Once the temperature of the CMA medium was around 40–45 °C, 2 mL of this stock solution was added [24,25]. In cases where there were no apparent mycelia on the seed surface but attached sporangia were visible under the microscope, each seed was deposited into the center of each Petri dish containing modified CMA medium. These Petri dishes were incubated at 25 °C for 2 to 6 days until the development of a fungal colony.

### 2.4. Morphological Identification

The identification of the pathogenic oomycetes was initially based on visual observation of the forming colony (color and shape), mycelial growth, and sporulation. Under the light microscope, colony and hyphal morphology, and the production and morphological traits of sporangia, oogonium, antheridia, oospores and chlamydospores were examined accordingly [4,26,27,28,29]. To induce the formation of sporangia, mycelial plugs of 1 cm in diameter were cut from the edges of 5-day old growing cultures on V8 agar medium and then placed in Petri dishes previously filled with 10 mL of non-sterile soil extract [4]. The Petri dishes were placed at 18 °C for 24 h followed by 4 °C for 2 h [30]. The dishes were examined daily for sporangial development and zoospore release over a period varying from 2 to 7 days.

### 2.5. Molecular Identification

Fresh mycelia were harvested by scraping the surface of 7-day old growing culture onto PDA medium with a sterile spatula using the protocol described by Doyle and Doyle [31] with some modifications. Approximately 50 to 100 mg of the mycelium of each fungal isolate were placed in 2 mL Eppendorf tubes containing 500 μL of the extraction buffer. The mixture was ground for 1 min, incubated for 30 min at 65 °C in a water bath and briefly centrifuged at 13,000 rpm for 5 min. Afterward, 400 μL of the supernatant was recovered and an equivalent volume (400 μL) of chloroform/isoamyl alcohol (24/1) was added. The mixture was gently vortexed for 5 min and centrifuged at 14,000 rpm for 5 min. The supernatant (350 μL) was recovered, precipitated with isopropanol (350 μL), and then centrifuged at 14,000 rpm for 10 min. The supernatant was then removed and 500 μL of ethanol at 70 °C was added to the pellet, vortexed, and centrifuged for 5 min at 14,000 rpm. The pellet was dried in an incubator at 60 °C (30–45 min), resuspended in 50 μL of SDW, and stored at −80 °C until use.

Molecular identification was performed by sequencing the Cox II gene using a pair of primers amplifying 563 bp of FM78 (5′-ACAAATTTCACTACATTGTCC-3′), and FM75 (5′-CCTTGGCAATTAGGATTTCAAGAT-3′), DNA amplification for the detection of *Phytopythium* species was carried as follow: one cycle at 95 °C for 2 min; followed by 35 cycles of 1 min annealing at 56 °C, 2 min extension at 72 °C, and 1 min denaturation at 94 °C; and finally by one extension cycle at 72 °C for 10 min [32]. The PCR amplified products were separated on 1% agarose gel in 1X TBE buffer (Tris-boric acid-EDTA), and stained with 0.4% Ethidium bromide, and visualized under UV light. The PCR products were sequenced at STAB Vida Inc. (Caparica, Portugal) using Sanger dideoxy sequencing method. The Cox II sequences of each isolate was compared to the sequences submitted to GenBank. The species identity of each fungal isolate was determined using Basic Local Alignment Search Tool (BLAST), where the analysis of each sequence is grouped with the most closely related species at NCBI-BLAST (http://www.ncbi.nlm.nih.gov/BLAST/, accessed on 1 July 2021).

Phylogenetic and molecular evolutionary analyses were conducted using MEGA version 10.1.7 [33]. An alignment of the Cox II sequences was generated using Clustal W [34]. Phylogenetic analyses of Cox II were performed by the maximum likelihood method and Kimura 2-parameter model [35]. The phylogenetic tree was evaluated by bootstrap analysis based on 1000 replicates. In this study, a closely related species of *Phytopythium* vexans (AB468910), *Pp. helicoides* (MW450816), *Pp. sindhum* (KJ595436), *Pp. litorale* (MT050458), *Pythium mercuriale* (AB920504), *Phytophthora tropicalis* (DQ469735), *Phytophthora capsici* (DQ469734), *Phytophthora lateralis* (AY129207), *Phytophthora palmivora* (AY129220), *Pythium ultimum* (AF196640), *Pythium aphanidermatum* (AB160854), *Pp. aichiense* (AB948192), *Pp. babaiiaharii* (MT720670), *Pp. boreale* (AB690677), *Pp. carbonicum* (AB690678), *Pp. chamaehyphon* (AB690674), *Pp. citrinum* (AB690679), *Pp. delawarense* (AB690672), *Pp. fagopyri* (AB690671), *Pp. iriomotense* (AB690689), *Pp. longitubum* (MT720672), *Pp. megacarpum* (AB690665), *Pp. montanum* (AB690667), *Pp. nanjigens* (MG788317), *Pp. oedochilum* (AB690676), and *Pythium oligandrum* (EU265664), were used as references.

### 2.6. Pathogenicity Test

In order to fulfill Koch’s postulates and to ensure that all fungal isolates were able to produce the disease, a pathogenicity test was performed on 115 one-year old apple trees as previously described [4]. Fungal isolates were grown on PDA at 25 °C for 7 days. Each apple plant was wounded at two sites, collar and stem, with a cork-borer at a diameter of 5 mm, each wound was filled with a mycelial plug (diameter 5 mm), cut from the edge of a fresh, actively growing colony of each fungal isolate and then covered with parafilm. Wounded plants inoculated with PDA free of fungal mycelia were used as controls. This experiment was repeated twice with four replicates for each fungal isolate. Inoculated and control plants were grown in pot cultures (20 cm diameter × 19 cm deep) containing a potting soil. All pots were arranged in full randomized design and watered once a week or more when needed. The water irrigation was applied directly to the soil. The greenhouse temperature was around 28 °C and maintained with heated ventilation. Plants were inspected weekly for symptoms. The onset of symptoms were recorded 4 months post-inoculation. At the end of the experiment, the size and color of the necrotic lesions induced by each fungal pathogen was recorded. Data collected included visual ratings of shoots and roots, as previously described by Rodriguez-Padron et al. [17]. Accordingly, shoots were visually inspected for disease symptoms and rated on a scale of 1 to 5 with 1 = healthy leaf, 2 = early yellowing, 3 = half-yellowed leaf 4 = dry leaf, and 5 = wilting leaf. Roots were cleaned, visually inspected for root rot and rated on a scale of 1 to 5 with 1 = healthy white roots/ no disease recovered; 2 = 25% root rot or seemingly healthy roots + onset of root rots), 3 = 50% root rot and early browning, 4 = 75% root rot (brown rot) and 5 = 100% dead roots-root system destroyed. Fungal isolates were re-isolated from necrotic tissues to complete Koch’s postulates.

### 2.7. Statistical Analysis

A chi-square analysis test (*χ*^2^) was used to assess the relationship between disease prevalence and some common farming practices on the Saïss plain, such as rootstocks, soil type, and type of plantation, cultivars, and irrigation mode in orchards. All tests were arranged in a completely randomized design (CRD). Analysis of variance (ANOVA) was performed using SPSS statistical software (IBM SPPSS Statistics 25) to assess the effect of isolate inoculation. When the effect was revealed to be significant, the least square difference (LSD) test was employed for mean separation (*p* < 0.05).

## 3. Results

### 3.1. Field Symptoms

Decline symptoms on trees were observed in pear and apple growing areas of the Fes-Meknès region of Morocco. These symptoms included a quick dieback, necrotic brown lesions in the bark, defoliation and yellowing, wilting, and root rot leading to the death of the infected tree (Figure 2). Accordingly, an extensive survey of 50 fields of apple and pear was performed in order to determine the causal agents of the tree decline in the region. After a deep investigation into the cultural practices adopted by the farmers, in particular excessive watering of trees, it was assumed that the disease was caused by oomycetes. Therefore, laboratory analyses were performed to look for the presence of these oomycete pathogens by using soil baiting.

### 3.2. Isolation, Morphological, and Molecular Identification of Fungal Isolates

**Figure 3:**
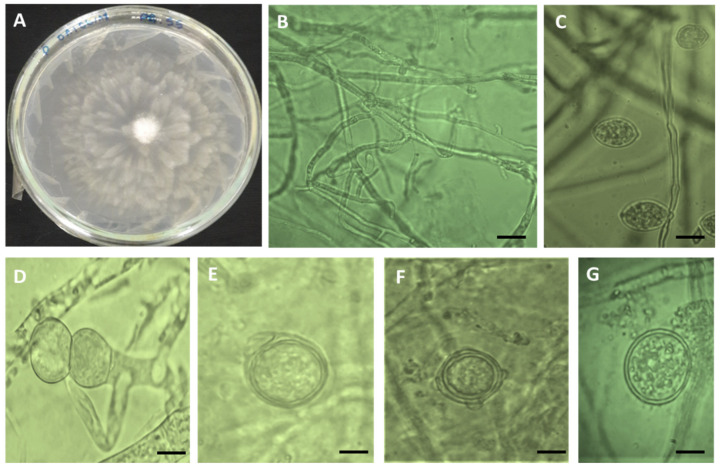
Morphology of *Phytopythium vexans*: white transparent colony on PDA medium (**A**), coenocytic hyphae (**B**), sporangia (**C**), double oospore (**D**), oogonium with single lobe branched antheridium (**E**), oogonium with two lobes branched antheridium (**F**), and thick-walled chlamydospores (**G**). Bar scale = 15 µm.

A total of 35 oomycetous isolates were obtained from representative samples of 50 surveyed fields (Table 1). The visual observation of morphological traits such as color and shape of the obtained growing colony underlines a typical colony of an oomycete pathogen (Figure 3). All fungal colonies had a whitish-blooming mycelium appearance with no visual sporulation at the surface, which confirmed their identity as oomycetous mycelia. Under the light microscope, non-septate hyphae were observed for all 35 fungal isolates with major hyphae ranging in width from 2.5 to 6.3 µm (Figure 3A). Furthermore, the microscope examination detected the presence of oomycete traits in V8 medium after incubation for 4 days, such as coenocytic hyphae (Figure 3B), globose/subglobose sporangia (Figure 3C) with/out papilla, 15.9 × 26.10 µm in diameter, double oospore (Figure 3D), oogonium with single lobed branched antheridium (Figure 3E), oogonium with two-lobed branched antheridium (Figure 3F), and thick-walled chlamydospores (Figure 3G). Oogonia were smooth, globose, and terminal with an average diameter of 26 µm. Zoospores released from sporangia were *Pythium*-like.

**Figure 4:**
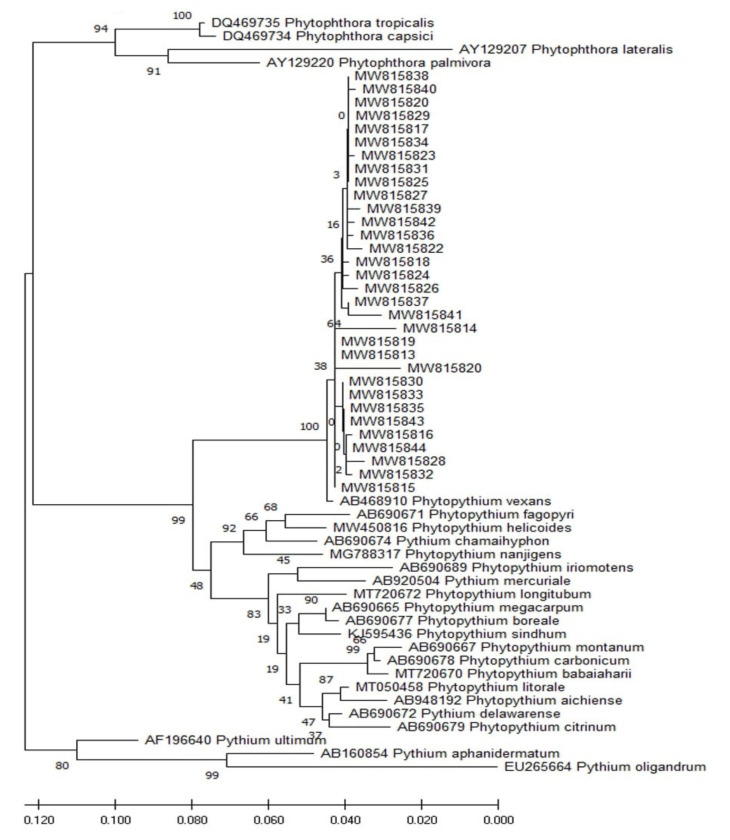
Phylogenetic tree of oomycetes fungi *Pp. vexans* was constructed using the maximum likelihood method on their cytochrone oxidase subunit II sequences, showing the position of our oomycetes isolates that were obtained in this study within the *Phytopythium* and P*ythium* complex. Isolates from this study are indicated in bold characters. The sequences of *Phytopythium*, *Phytophthora* and *Pythium* from Genebank; *Pp. vexans (*AB468910*)*, *Pp. helicoides (*MW450816*)*, *Pp. sindhum (*KJ595436*)*, *Pp. litorale (*MT050458*)*, *Pythium mercuriale (*AB920504*)*, *Phytophthora tropicalis (*DQ469735*)*, *Phytophthora capsici (*DQ469734*)*, *Phytophthora lateralis (*AY129207*)*, *Phytophthora palmivora*, *(*AF196640*) Pythium ultimum (*AF196640*)*, *Pythium aphanidermatum (*AB160854*)*, *Pp. aichiense (*AB948192*)*, *Pp. babaiiaharii (*MT720670*)*, *Pp. boreale (*AB690677*)*, *Pp. carbonicum (AB690678)*, *Pp. chamaehyphon (AB690674)*, *Pp. citrinum (AB690679)*, *Pp. delawarense (*AB690672*)*, *Pp. fagopyri (*AB690671*)*, *Pp. iriomotense (*AB690689*)*, *Pp. longitubum (*MT720672*)*, *Pp. megacarpum (*AB690665*)*, *Pp. montanum (*AB690667*)*, *Pp. nanjigens (*MG788317*)*, *Pp. oedochilum (*AB690676*)*, and *Pythium oligandrum (*EU265664*)*, were used as references. Numbers at the nodes of clusters represent the bootstrap values that were generated from 1000 pseudoreplicates.

To confirm the exact identity of the obtained oomycetous isolates, specific primers aiming to identify the cytochrome subunit II (Cox II) gene were used. Interestingly, the results of sequencing showed that all isolates were 99% similar to those of *Pp. vexans* in the genbank. Results from the blast and phylogenetic tree analysis of Cox II showed the high similarity of the isolates to *Pp. vexans*; the phylogenic analysis grouped all the isolates into a single clade of *Pp. vexans* (Figure 4, Table 1).

### 3.3. Distribution of the Pathogenic Oomycetes in the Surveyed Apple and Pears Orchards

The prevalence of oomycetes in samples taken from each region was shown in Table 2. Results indicated that positive samples 20, 8, 3, 2, and 2 were from the Imouzzer, Sefrou, Azrou, El Hajeb, and Meknes locations, respectively. Moreover, no samples from Sidi Lmakhfi district in Azrou and Chlihat district in El-Hajeb were revealed to be positive for the pathogen (Table 1 and Table 2). However, the Imouzzer location was the most infected of all the surveyed regions.

**Figure 5:**
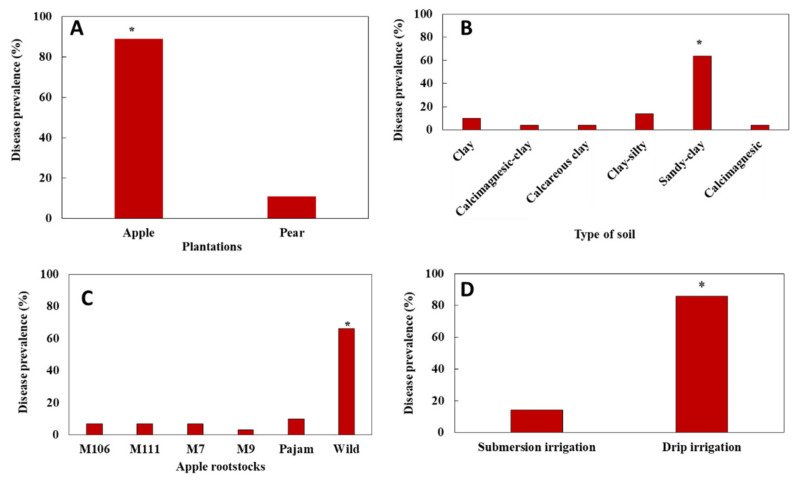
Disease prevalence (%) according to the type of plantation apple/pear tree (**A**), type of soil (**B**), apple rootstocks (**C**), and irrigation mode (submersion versus drip irrigation mode) in the surveyed orchards of Saïss plain (**D**). Asterisks (*) indicate significant effect (*p* < 0.05) according to the Chi square analysis test (*χ*^2^).

The survey pointed out that similar symptoms occurred on apple and pear trees, with a higher frequency on apple trees (Table 1 and Figure 5A). Results also indicated that there was a significant effect (*p* < 0.05) of soil type on disease prevalence (Figure 5B). A significant effect was also seen with rootstocks (*p* < 0.028). The wild rootstock was the most susceptible to the disease in comparison with the other rootstocks (Figure 5C).

Furthermore, results pointed out that drip irrigation (75%) versus submersion irrigation was the most used irrigation mode in the prospected region with an average flow rate of 16 L/h per tree. The duration of irrigation ranged from 1–2 h with an interval of 24 to 48 h and differed among farmers and according to season. In contrast, the amount of water delivered by submersion irrigation ranged from 80 to 100 L per tree with an interval of 5 days to one week between two successive watering’s. Furthermore, the impact of the watering system on disease prevalence was significant (*p* < 0.05). The highest prevalence was observed on farms adopting drip irrigation (Figure 5D). This result was considered normal as most of the farms surveyed adopted drip irrigation.

### 3.4. Pathogenicity Test

**Figure 6:**
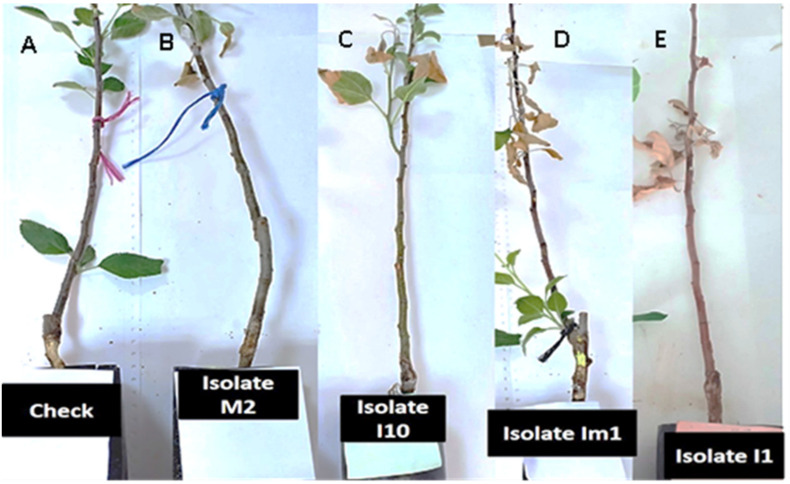
Symptoms of yellowing, wilting, and lesions on stems of apple rootstock M115 seedlings at the end of the stem pathogenicity test: healthy control ((**A**), uninoculated plants); plants inoculated with *Phytopythium vexans* M2 (**B**), *Pp. vexans* I10 (**C**), *Pp. vexans* Im1 (**D**), and *Pp. vexans* I1 (**E**).

**Figure 7:**
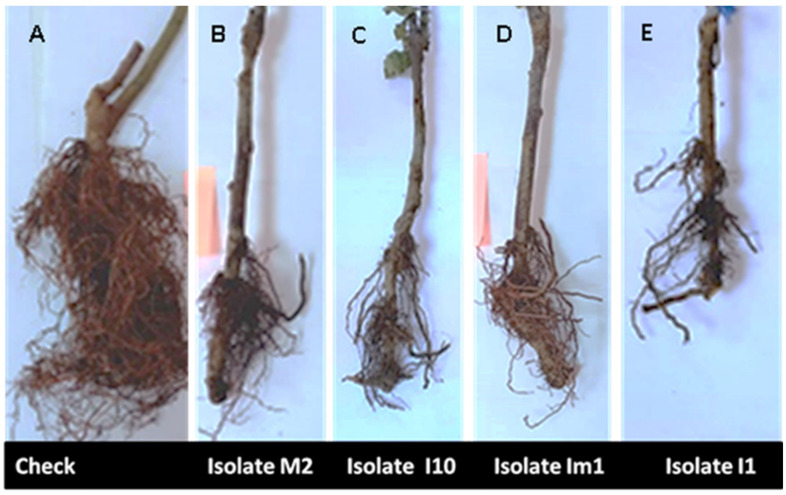
Root rot symptoms in apple rootstock M115 seedlings at the end of the root pathogenicity test (four months post-inoculation) under greenhouse conditions: healthy control ((**A**), uninoculated plants); plants inoculated with *Phytopythium vexans* M2 (**B**), *Pp. vexans* I10 (**C**), *Pp. vexans* Im1 (**D**), and *Pp. vexans* I1 (**E**).

All fungal isolates were pathogenic to apple seedlings and most showed symptoms 1-month post-inoculation. Accordingly, necrotic lesions on the inoculated stem were observed, for the first time, 18 days post-inoculation, and after 30 days post-inoculation, more than 95% of the inoculated apple seedlings showed symptoms most likely similar to those observed in the field (Figure 6). The bark tissue turned brown and rotted, and the lesion reached the wood, while control plants were healthy and asymptomatic. Four months later, apple seedling growth was substantially reduced compared to un-inoculated seedlings, which were healthy and grew normally and the root system of inoculated seedlings presented significant necrosis and acute decay (Figure 7). All inoculated pathogens were successfully re-isolated from the inoculated stems and collars of apple seedlings.

Results of pathogenicity tests after 4 months post-inoculation are listed in Table 3. Statistical analysis showed a highly significant effect (*p* < 0.0001) of fungal isolates on collar and stem lesion length, leaves, and root necrosis indices relative to untreated controls (Table 3). In addition, a significant difference was observed between *Pp. vexans* isolates for all evaluated traits. *Pp. vexans* isolates I17, I3, I1, and I12 were revealed to be the most aggressive with recorded lesion sizes (cm) of 5.46 ± 0.11, 4.53 ± 0.55, 6.13 ± 0.15, 6.12 ± 0.75, respectively. These isolates induced a marked dryness on the stem. On the collar, results underlined that isolate S5 was the most aggressive and had a lesion size of 5.93 ± 0.15 (Table 3). Furthermore, disease symptoms on leaves and roots were rated between 4 and 5 and were statistically different from the un-inoculated control plants (*p* < 0.0001). Significance differences were also observed between oomycete isolates for these two evaluated parameters (Table 3). For all isolates, root rot disease severities ranged from 70% to 100%.

## 4. Discussion

In recent years, symptoms of decline such as root rot, brown rot, and crown canker have been observed on pear and apple fruit trees in the Saiss region, causing serious damage and reducing overall yield. In this study, we confirmed through morphological and molecular characterization, and pathogenicity tests that these symptoms of decline were mainly due to *Pp. vexans.* This result is in agreement with our previous findings [21] in which *Pp. vexans* was the main pathogen of apple dieback in the Saïss region. The pathogen *Pp. vexans*, which threatens apple and pear trees in particular, was found in 34 of 50 samples from different sites of commercial apple and pear orchards in the Fez-Meknes region.

The pathogen, *Phytopythium* is considered to be an intermediate between *Phytophthora* and *Pythium* species [36]. It is a new genus of the Pythiaceae family and the order Peronosporales, which was described with *Phytopythium sindhum* as a type species by [37]. The pathogenic oomycete *Pp. vexans* was reported on apple trees in South Africa in 2011 [5] and on citrus in China [38] and Tunisia in 2017 [15]. It was also isolated from infected avocado trees in the Canary Islands [17], on avocado in Mexico [39], on kiwi in Turkey [16], on grapevines in South Africa [19], on cassava in Brazil [40], and in Vietnam on durian [41]. Furthermore, the analysis of the phylogenetic tree underlined a strong similarity of our isolates of *Pp. vexans* with those of different countries mentioned above. Under the light microscope, *Pp. vexans* colonies appeared more similar to *Pythium* than *Phytophthora*, while symptoms observed in the field as well as those obtained from the pathogenicity test of this study revealed a substantial similarity to those of *Phytophthora* diseases. It was concluded that both pathogens (*Phytopythium* and *Phytophthora*) cause root rot, collar and crown canker, branching of roots, yellowing of leaves followed by wilting, necrotic bark becoming dry, and brown lesions in the neck [42]. It was noted that all of these symptoms were observed and recorded during the field and pathogenicity test. In addition, *Phytophthora* and *Phytopythium* are able to grow at variable temperatures [43], so it will be useful to study the influence of temperature on the growth of *Pp. vexans*. Such a study can determine whether changes in environmental conditions are the cause of its occurrence in recent years.

Many hypotheses suggest that the main cause of apple and pear decline was excessive irrigation, as these kinds of pathogens require high moisture for their proliferation, production of sporangia, and release of infectious propagules and zoospores [44]. Therefore, it was concluded that irrigation may have induced *Pp. vexans* infection on apple trees, particularly in orchards with a high planting density [45]. In addition, Moein et al. [46] indicated that the use of higher irrigation regimes was likely the cause of the greater disease severity on inoculated apple seedlings with either *P. ultimum*, *P. irregulare*, *P. sylvaticum*, *Pp. vexans,* or *Phytophthora cactorum*. Therefore, any action aiming to prevent a humid microclimate in the cover will constitute an element of management since this disease develops more rapidly under these conditions [47]. Our results pointed out that the disease was more frequent in drip irrigation than in submersion irrigation due to improper management of irrigation systems by farmers who used drippers with a high-rate (16 l/h/tree) instead of those with low flow rates (8 l/h/tree). These results were in complete agreement with those reported by Benfradj et al. [15] who found that *Pp. vexans* disease was more prevalent in drip irrigation. In our study, the disease was found in drip and in submersion irrigation as well, however, its prevalence was higher as most farmers opted for drip irrigation with a higher flow rate and long duration. Therefore, it is important to manage the water supply as closely as possible to the needs of the crop and to irrigate by drip rather than by submersion. Water in its various forms is one of the vectors of oomycetes, which could be considered the most important mode of dissemination. Indeed, they produce motile flagellated zoospores in water, which attack the trees through the roots. Thus, irrigation using contaminated water infects seedlings by *Phytophthora alni* in the nursery [48,49]. In addition, the reuse of collected irrigation water is a practice presenting a high risk in the nursery [50]. Without the installation of an effective disinfection system, these waters can cause epidemics on irrigated plants. Depending on how it works, water can spread infectious spores over varying distances, and a single drop of rainwater is capable of disseminating spores in a radius of one meter [45].

The importance of the different propagules of oomycete fungi in the development of their respective diseases differs according to the mechanism of dispersion. In the case where the dispersion is carried out by free water, it is the zoospores that are involved in the proliferation of the oomycete [51,52]. Regardless of the dispersal mechanism, zoospores are continuously important in most oomycetous species, because they must swim and locate the roots [53]. However, some *Pythium* species do not produce zoospores, which means that there is another dispersal mechanism other than irrigation water [54]. Carlile et al. [55] pointed out that zoospores represent 95% of the *Phytophthora* propagules recovered from irrigation water. They explained this finding to be due to their ability to swim, while other propagules (the mycelium, chlamydospores, etc.) tend to sink to the bottom of the water [56]. Other methods of dispersing oomycete propagules in fields and orchards include movement of infested soil, infested nursery stock, and infested dust particles, all containing mycelium, chlamydospores, or oospores [57]. These findings can explain the highest level of infected samples in Imouzzer compared with other locations due to excessive watering by small farmers as most of them are not yet familiarized with drip irrigation.

The results of this study indicated that the prevalence of decline disease varies with respect to the soil type and cultivar. It was found that the disease was more pronounced in sandy-clay soil than in other types of soil. This might be due to the ability of zoospores to move within soil particles and reach their root targets in a short amount of time. Moein et al. [47] pointed out that soil temperature and soil type are primary factors in the interaction of oomycetes with their plant hosts and the subsequent severity of infection was often dependent on these factors. Therefore, high disease severities were reported in clay soils due to favourable conditions for the dispersal of zoospores facilitated by the high water holding capacity of this type of soil [52,58]. Furthermore, serious damages by some *Pythium* species were found in warmer areas, whereas other *Pythium* species are more prevalent and virulent at lower temperatures [59,60].

Managing root rot of rosaceous trees is a difficult task and requires the use of an integrated pest management (IPM) approach, as no single control strategy will prevent or control this disease. However, systemic chemicals such as phenylamides and fenamiphos were shown effective for managing rot root diseases caused by the oomycete group [61]. The effectiveness of some phosphonates and phenylamides, such as mefenoxam and metalaxyl, was demonstrated against *Phytophthora* diseases under field conditions [47]. Furthermore, several *Pythium* species associated with root rot diseases of carrot, soybean, corn, and forest nurseries were shown to be sensitive to metalaxyl, mefenoxam, and fosetyl-Al [62,63,64]. However, Matthiesen et al. [64] noticed that the aggressiveness and fungicide sensitivity of *Pythium* spp. was significantly affected by the temperature. Similar active substances were used against *Phytopyhtium* spp. on soybean and corn [65]. In Morocco, the control of root rot of trees caused by oomycetes relies on the use of fungicides as trunk injections with phosphonates or in application with drip watering. Soil fungicide applications, including subsurface drip chemigation, have recently gained interest as a method of improving control *Phytophthora* crown and root rot [66].

## 5. Conclusions

This study confirmed our previous findings and highlighted that *Pp. vexans* was the major cause of apple and pear decline in Morocco. It was concluded that irrigation mode, rootstock, and type of soil significantly impacted the disease. Therefore, the epidemiological and economic relevance of these oomycetes as a causal agent of apple and pear diseases in commercial orchards in Morocco deserves further investigation. Accordingly, different strategies to control this oomycetous pathogen and its subsequent disease are being evaluated and implemented. The results of this study could help to better understand the frequent occurrence of the disease and to develop reliable integrated strategies for its management that take into account the use of rational irrigation, appropriate chemicals, and sanitation practices.

## Figures and Tables

**Table 1:** Identity of *Phytopythium vexans* isolates based on morphology and COX II genes, and their GenBank accessions numbers (an.).

Fungal Isolate	Origin	Type of Plantation	Rootstock	Type of Soil	Identified Species	Genbank COX II an. ^1^
E4	El-Hajeb	Pear tree	M106	Clay	*Phytopythium vexans*	MW815816
E5	El-Hajeb	Apple tree	Pajam	Clay	*Phytopythium vexans*	-
M1	Meknès	Apple tree	M111	Calcimagnesic	*Phytopythium vexans*	MW815828
M2	Meknès	Apple tree	Pajam	Calcimagnesic	*Phytopythium vexans*	MW815829
S1	Sefrou	Pear tree	Wild	Calcimagnesic-clay	*Phytopythium vexans*	MW815836
S2	Sefrou	Pear tree	Wild	Calcimagnesic-clay	*Phytopythium vexans*	MW815837
S3	Sefrou	Apple tree	M7	Sandy-clay	*Phytopythium vexans*	MW815838
S4	Sefrou	Apple tree	M9	Sandy-clay	*Phytopythium vexans*	MW815839
S5	Sefrou	Apple tree	M7	Sandy-clay	*Phytopythium vexans*	MW815840
S8	Sefrou	Apple tree	M9	Sandy-clay	*Phytopythium vexans*	MW815841
S9	Sefrou	Apple tree	M9	Sandy-clay	*Phytopythium vexans*	MW815842
S10	Sefrou	Apple tree	M7	Sandy-clay	*Phytopythium vexans*	MW815843
A1	Azrou	Apple tree	M9	Calcareous-clay	*Phytopythium vexans*	MW815813
A6	Azrou	Apple tree	Wild	Clay-silty	*Phytopythium vexans*	MW815814
A7	Azrou	Apple tree	Pajam	Clay-silty	*Phytopythium vexans*	MW815815
I1	Imouzzer	Apple tree	Wild	Sandy-clay	*Phytopythium vexans*	-
Im1	Imouzzer	Apple tree	Pajam	Sandy-clay	*Phytopythium vexans*	MW815817
I2	Imouzzer	Pear tree	Wild	Sandy-clay	*Phytopythium vexans*	-
I3	Imouzzer	Apple tree	Wild	Sandy-clay	*Phytopythium vexans*	MW815819
Im3	Imouzzer	Apple tree	Wild	Sandy-clay	*Phytopythium vexans*	MW815818
I5	Imouzzer	Apple tree	M106	Sandy-clay	*Phytopythium vexans*	MW815830
I6	Imouzzer	Apple tree	M111	Sandy-clay	*Phytopythium vexans*	MW815831
I7	Imouzzer	Apple tree	Wild	Sandy-clay	*Phytopythium vexans*	MW815832
I8	Imouzzer	Apple tree	Wild	Sandy-clay	*Phytopythium vexans*	MW815833
I10	Imouzzer	Apple tree	Wild	Sandy-clay	*Phytopythium vexans*	MW815834
I12	Imouzzer	Apple tree	Wild	Sandy-clay	*Phytopythium vexans*	MW815825
I13	Imouzzer	Apple tree	Wild	Sandy-clay	*Phytopythium vexans*	MW815835
I14	Imouzzer	Apple tree	Wild	Sandy-clay	*Phytopythium vexans*	MW815844
I15	Imouzzer	Apple tree	Wild	Sandy-clay	*Phytopythium vexans*	MW815820
I16	Imouzzer	Apple tree	Wild	Sandy-clay	*Phytopythium vexans*	MW815821
I17	Imouzzer	Apple tree	Wild	Sandy-clay	*Phytopythium vexans*	MW815822
I18	Imouzzer	Apple tree	Wild	Sandy-clay	*Phytopythium vexans*	MW815823
I19	Imouzzer	Apple tree	Wild	Sandy-clay	*Phytopythium vexans*	MW815824
I20	Imouzzer	Apple tree	Wild	Sandy-clay	*Phytopythium vexans*	MW815826
I21	Imouzzer	Apple tree	Wild	Sandy-clay	*Phytopythium vexans*	MW815827

^1^ X: a total of 35 isolates of *Phytopythium vexans* were identified morphologically and based on phylogenetic tree analyses and had their Cox II gene subsequently sequenced and deposited in Genebank. (-): missed sequences.

**Table 2:** Occurrence and distribution of pathogenic oomycetes in apple and pear orchards in different regions in Morocco.

Location	District	No. Orchards ^a^	Positive Isolations	
			*Phytopythium vexans*	Disease Prevalence (%)
Azrou	Tigrigra	2	1	33
	Sidi Lmakhfi	2	-	
	Ain Louh	5	2	
El-Hajeb	Tamchachate	3	2	40
	Chlihat	2	-	
Imouzzer	Ain Chifa	9	8	83
	Aït Sbaà	14	11	
	Farha	1	1	
Meknès	Majjat	2	2	100
Sefrou	Aghbalou Akourar	2	2	80
	Laanoucer	8	6	
Total		50	35	70

^a^ Numbers of surveyed orchards.

**Table 3:** Results of pathogenicity tests in apple seedlings showing the averaged collar and stem lesion length (cm), leaves, and root necrosis indices (1–5) after 4 months post-inoculation.

Fungal Isolates	Lesion Length (cm)		Scale Severity Symptoms	
	Collar	Stem	Leaves Wilting ^2^	Roots Necrosis ^3^
*Pp. vexans* E4	4.20 ^1^ ± 0.1 ^ghij^	2.93 ± 0.15 ^c^	1.00 ± 0.00 ^b^	4.66 ± 0.57 ^cd^
*Pp. vexans* E5	3.60 ± 0.15 ^cd^	3.06 ± 0.11 ^cd^	1.00 ± 0.00 ^b^	3.66 ± 0.57 ^b^
*Pp. vexans* M1	5.06± 0.05 ^opq^	3.57 ± 0.57 ^efgh^	1.66 ± 0.57 ^bcd^	4.00 ± 1.00 ^bc^
*Pp. vexans* M2	3.60 ± 0.10 ^cd^	3.03 ± 0.05 ^cd^	2.00 ± 0.00 ^cde^	4.33 ± 0.57 ^bcd^
*Pp. vexans* S1	4.53 ± 0.05 ^jklm^	3.33 ± 0.11 ^cdef^	1.33 ± 0.57 ^bc^	4.33 ± 0.57 ^bcd^
*Pp. vexans* S2	4.56 ± 0.11 ^klm^	3.80 ± 0.10 ^ghij^	2.33 ± 0.57 ^def^	4.66 ± 0.57 ^cd^
*Pp. vexans* S3	3.93 ± 0.15 ^defg^	4.10 ± 0.10 ^ijk^	2.00 ± 0.00 ^cde^	5.00 ± 0.00 ^c^
*Pp. vexans* S4	4.8 ± 0.10 ^mno^	3.6 0± 0.26 ^efgh^	5.00 ± 0.00 ^l^	4.66 ± 0.57 ^cd^
*Pp. vexans* S5	5.93 ± 0.15 ^t^	4.20 ± 0.10 ^jk^	4.66 ± 0.57 ^kl^	4.66 ± 0.57 ^cd^
*Pp. vexans* S8	5.16 ± 0.15 ^pqr^	3.70 ± 0.10 ^fghi^	3.00 ± 0.00 ^fgh^	4.66 ± 0.57 ^cd^
*Pp. vexans* S9	4.40 ± 0.10 ^ijkl^	3.40 ± 0.10 ^defg^	2.66 ± 0.57 ^efg^	5.00 ± 0.00 ^c^
*Pp. vexans* S10	4.96 ± 0.11 ^nop^	3.43 ± 0.57 ^defg^	4.67 ± 0.57 ^kl^	4.66 ± 0.57 ^cd^
*Pp. vexans* A1	3.50 ± 0.52 ^bc^	3.36 ± 0.49 ^cdefg^	1.33 ± 0.57 ^bc^	4.66 ± 0.57 ^cd^
*Pp. vexans* A6	3.60 ± 0.10 ^cd^	3.10 ± 0.10 ^cd^	1.00 ± 0.00 ^b^	4.33 ± 0.00 ^bcd^
*Pp. vexans* A7	4.46 ± 0.32 ^ijklm^	3.62 ± 0.15 ^efgh^	1.00 ± 0.00 ^b^	4.66 ± 0.57 ^cd^
*Pp. vexans* I1	5.56 ± 0.60 ^s^	6.13 ± 0.15 ^m^	4.33 ± 0.57 ^jkl^	4.33 ± 0.57 ^bcd^
*Pp. vexans* Im1	4.30 ± 0.20 ^hijk^	4.00 ± 0.10 ^hij^	4.66 ± 0.57 ^kl^	4.66 ± 0.57 ^cd^
*Pp. vexans* I2	5.10 ± 0.20 ^opqr^	4.53 ± 0.30 ^k^	2 ± 1.00 ^cde^	4.66 ± 0.57 ^cd^
*Pp. vexans* I3	4.67 ± 0.15 ^lmn^	7.23 ± 0.55 ^n^	2.33 ± 0.57 ^def^	5.00 ± 0.00 ^c^
*Pp. vexans* Im3	5.36 ± 0.32 ^qrs^	4.06 ± 0.11 ^ij^	4.00 ± 0.00 ^ijk^	5.00 ± 0.00 ^c^
*Pp. vexans* I5	4.16 ± 0.11 ^ghi^	3.80 ± 0.1 ^ghij^	1.66 ± 0.57 ^bcd^	4.66 ± 0.57 ^cd^
*Pp. vexans* I6	3.76 ± 0.25 ^cdef^	3.06 ± 0.11 ^cd^	1.00 ± 0.00 ^b^	5.00 ± 0.00 ^c^
*Pp. vexans* I7	5.03 ± 0.15 ^opq^	2.13 ± 0.15 ^b^	4.00 ± 0.00 ^ijk^	4.33 ± 0.57 ^bcd^
*Pp. vexans* I8	4.00 ± 0.10 ^efgh^	3.23 ± 0.25 ^cde^	5.00 ± 0.00 ^l^	4.33 ± 0.57 ^bcd^
*Pp. vexans* I10	4.80 ± 0.20 ^mno^	3.68 ± 0.05 ^fghi^	3.00 ± 0.00 ^fgh^	4.66 ± 0.57 ^cd^
*Pp. vexans* I12	4.66 ± 0.41 ^lmn^	6.20 ± 0.75 ^m^	3.66 ± 0.57 ^hij^	5.00 ± 0.00 ^c^
*Pp. vexans* I13	3.93 ± 0.15 ^defg^	3.70 ± 0.10 ^fghi^	5.00 ± 0.00 ^l^	5.00 ± 0.00 ^c^
*Pp. vexans* I14	4.03 ± 0.05 ^fgh^	3.26 ± 0.28 ^cdef^	1.00 ± 0.00 ^b^	4.66 ± 0.57 ^cd^
*Pp. vexans* I15	5.40 ± 0.15 ^rs^	3.20 ± 0.20 ^cde^	4.66 ± 0.57 ^kl^	5.00 ± 0.00 ^c^
*Pp. vexans* I16	3.93 ± 0.15 ^defg^	3.30 ± 0.20 ^cdef^	5.00 ± 0.00 ^l^	4.33 ± 0.57 ^bcd^
*Pp. vexans* I17	3.23 ± 0.15 ^b^	5.46 ± 0.11 ^l^	3.33 ± 0.57 ^ghi^	4.33 ± 0.57 ^bcd^
*Pp. vexans* I18	4.73 ± 0.15 ^mno^	3.70 ± 0.10 ^fghi^	4.66 ± 0.57 ^kl^	4.66 ± 0.57 ^cd^
*Pp. vexans* I19	4.30 ± 0.20 ^hijk^	3.70 ± 0.20 ^fghi^	5.00 ± 0.00 ^l^	4.66 ± 0.57 ^cd^
*Pp. vexans* I20	3.66 ± 0.15 ^cde^	3.40 ± 0.25 ^defg^	3.00 ± 0.00 ^fgh^	4.00 ± 0.00 ^bc^
*Pp. vexans* I21	4.16 ± 0.15 ^ghi^	5.23 ± 0.68 ^l^	4.00 ± 1.00 ^ijk^	4.33 ± 0.57 ^bcd^
Un-inoculated control	0.00 ^a^	0.00 ^a^	0.00 ^a^	0.00 ^a^

^1^: Data are the average of two experiments with four replicates; in each column, values having the same letter are not significantly different according to the LSD test (*p* < 0.05). ^2^: Leaf wilting were rated on a scale 1 to 5. ^3^: Root rot was rated on a scale 1 to 5, with 1: no disease recovered; 2: 25% rot root; 3: 50% root rot; 4: 75% root rot and 5: 100% dead roots-root system destroyed.

## Data Availability

Data is contained within the article.
